# Gas fermentation – a biotechnological solution for today's challenges

**DOI:** 10.1111/1751-7915.12431

**Published:** 2016-10-28

**Authors:** Peter Dürre

**Affiliations:** ^1^ Institut für Mikrobiologie und BiotechnologieUniversität UlmUlmGermany

Costs for biotechnological processes in general are largely dictated by substrate prices. Thus, especially production of bulk chemicals from sugar or molasses suffers economically, if these compounds increase in price. A fairly recent solution to deal with this problem is the use of cheap gases as a carbon and energy source. Both, aerobic and anaerobic gas fermentations have meanwhile reached commercial level (Dürre and Eikmanns, 2015). Additional benefits are the use of non‐food feedstocks and the reduction of greenhouse gas emissions, if sustainable compounds are produced. Anaerobic gas fermentation is a preserve of autotrophic acetogenic bacteria, mostly of the genus *Clostridium*. Typical representatives are *Clostridium ljungdahlii* and *C. autoethanogenum* that can use pure CO or syngas (a mixture of mostly CO and H_2_) as carbon and energy sources. Such gas mixtures are also waste emissions from steel manufacture and chemical production lines. They can easily be obtained from gasification of biomass, too. Metabolic engineering already allowed a large expansion of the product portfolio of these bacteria, which will offer alternatives for replacement of current crude oil‐based chemical manufacturing processes. Commercial size plants are under construction in both Asia (e.g. in China Shougang Group and LanzaTech) and Europe (in Belgium ArcelorMittal and LanzaTech), situated within steel mills and operating by using the steel mill waste gases to produce ethanol. In addition, further processes have been developed for synthesis of jet fuel (press release of September 14th, 2016; http://www.lanzatech.com/low-carbon-fuel-project-achieves-breakthrough/). Pathways for commodity and specialty chemicals have already been implemented in *C. ljungdahlii* and *C. autoethanogenum*, such as e.g. butanol, acetone, and isopropanol (Köpke *et al*., 2010; Köpke and Liew, 2012; Simpson *et al*., 2012). Even CO_2_ can be used as a carbon source, if additional reducing power (e.g. H_2_) is provided. As an example, *Acetobacterium woodii* has been metabolically engineered to produce acetone in addition to acetate (Hoffmeister *et al*., 2016). Thus, we can envision a near future with carbon capture from waste gases for the production of hydrocarbon fuels and chemical building blocks. Having said this, I want, however, to emphasize what challenges still lie ahead.

One of the major problems in anaerobic gas fermentation is the severe energy limitation that acetogens have to deal with. The central metabolic route for CO or CO_2_ fixation is the Wood‐Ljungdahl pathway (Fig. 1A). One ATP is produced by the acetate kinase reaction, which, however, is consumed by the activation of formate to formyltetrahydrofolate. Thus, the only ATP left for growth and metabolism is generated via ion gradients and ATPase. The best‐studied ion pump is the so‐called Rnf complex, which can export either protons or sodium ions by oxidizing reduced ferredoxin and transferring the electrons to NAD^+^, thus generating NADH (Fig. 1B) (Biegel and Müller, 2010; Tremblay *et al*., 2013; Schuchmann and Müller, 2014). Especially thermophilic acetogens instead express an energy‐conserving hydrogenase (Ech), which generates a proton gradient by oxidizing reduced ferredoxin and transferring the electrons to protons, thus forming hydrogen (Fig. 1C). Finally, an electron‐transport chain involving menaquinone and cytochromes might be operating in *Moorella thermoacetica*, generating a proton gradient (Das and Ljungdahl, 2003; Schuchmann and Müller, 2014; Poehlein *et al*., 2015). A simultaneous presence of such systems in a single organism has so far only been shown for *M. thermoacetica*, but yet without any evidence for simultaneous expression and function. A parallel operation might be a way to overcome the energy barrier, which limits production of compounds with a high ATP demand. Analysis of the proposed electron‐transport chain in *Moorella*, in addition to well‐established biochemical technology, will be further facilitated by the meanwhile well advanced repertoire of genetic techniques for anaerobic *Firmicutes* (Huang *et al*., 2016; Minton *et al*., 2016). These will also allow to construct specific mutants, either inactivating or adding a specific system. If successful, this will as well have enormous consequences for industrial application, as, for example, the synthesis of isoprene requires 3 acetyl‐CoA and 3 ATP, which the metabolism of acetogens simply cannot afford yet. In addition to the ion pumps mentioned above, increased energy efficiency might also be achieved by other systems. A recent poster presentation reported the presence of an arginine deiminase pathway in *C. autoethanogenum* that allows additional ATP formation with arginine as a nitrogen source (Valgepea *et al*., 2016). So, it seems like a safe bet to predict that we will see exciting new findings on the energetics of this fascinating group of organisms.

**Figure 1:**
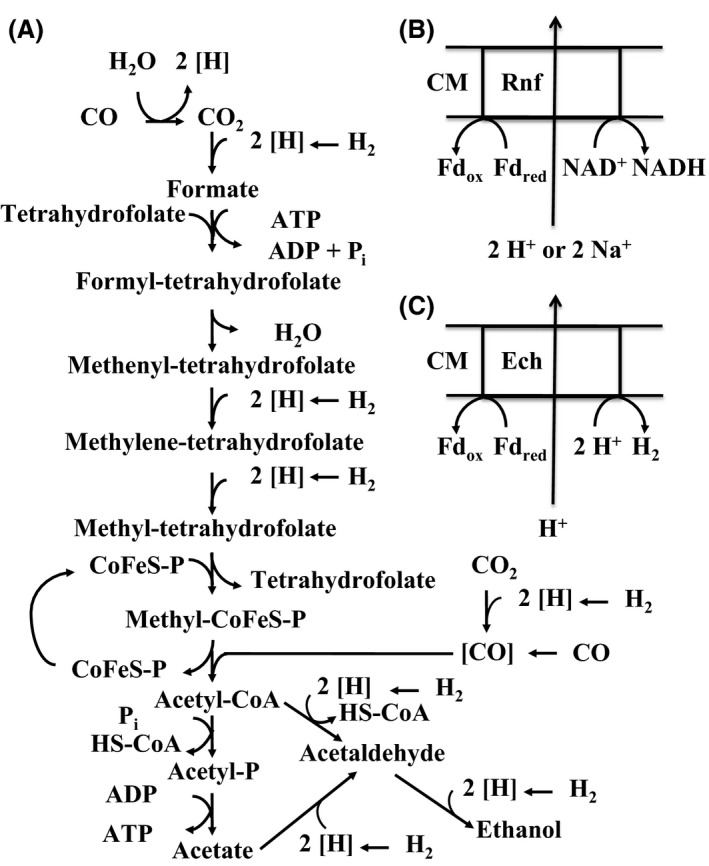
Schematic presentation of central metabolic reactions in autotrophic acetogens. (A) Wood‐Ljungdahl pathway, (B) Rnf membrane complex, (C) Ech membrane complex. 2 [H], reducing equivalents; P_i_, inorganic phosphate; CoFeS‐P, corrinoid iron‐sulphur protein; [CO], enzyme‐bound carbon monoxide; ‐P, phosphoryl group; HS‐CoA, coenzyme A; CM, cytoplasmic membrane; Fd_ox_, oxidized ferredoxin; Fd_red_, reduced ferredoxin.

Another major topic probably went by the scientific community without receiving much attention, because it is an issue that was brought up by lawyers. Probably all of us think that any product made by a living organism can be called ‘bio’. So, fuels made by microorganisms should be referred to as ‘biofuels’. However, this does currently not apply to the autotrophic acetogens. In Europe, the Renewable Energy Directive specifies the feedstocks that fuels can be made from, rather than the living organisms performing the reaction. So, if CO_2_ is stemming from biomass gasification, the product would qualify as a biofuel, if the CO_2_ is stemming from an industrial process (steel mill, chemical plant), it would not. This becomes critical when you see the financial benefits of qualifying under today's biofuels legislation, both in Europe and the US. Companies that do not fit in the regulations will have no market to sell their fuels. That in turn will of course affect funding opportunities for research, either directly from companies or from industrial participation in e.g. EU funding programmes, and, in the long run, will endanger this promising decarbonizing technology. Therefore, scientists need to emphasize that such misleading regulations must be corrected, in scientific publications as well as in information to politicians.

## Conflict of interest

None declared.
